# Efficacy of a Novel Respiratory Motion Reduction Block in Reducing Motion Artifact on Myocardial Perfusion Single-Photon Emission Computed Tomography

**DOI:** 10.7759/cureus.60656

**Published:** 2024-05-20

**Authors:** Hajime Ichikawa, Toyohiro Kato, Hiroki Kondo, Hideki Shimada, Takayuki Shibutani, Masahisa Onoguchi

**Affiliations:** 1 Department of Radiology, Toyohashi Municipal Hospital, Toyohashi, JPN; 2 Department of Quantum Medical Technology, Kanazawa University, Kanazawa, JPN; 3 Department of Cardiology, Nagoya University Hospital, Nagoya, JPN

**Keywords:** nuclear medicine, respiratory motion reduction, motion artifacts, respiratory motion, myocardial perfusion, single-photon emission computed tomography (spect)

## Abstract

Purpose: Motion artifacts caused by heart motion during myocardial perfusion single-photon emission computed tomography (SPECT) can compromise image quality and diagnostic accuracy. This study aimed to evaluate the efficacy of the novel respiratory motion reduction block (RRB) device in reducing motion artifacts by compressing the hypochondrium and improving SPECT image quality.

Methods: In total, 91 patients who underwent myocardial perfusion SPECT with ^99m^Tc-sestamibi were retrospectively analyzed. Patients (n = 28) who underwent SPECT without the RRB were included in the control group, and those (n = 63) who underwent SPECT with the RRB were in the RRB group. The distance of heart motion during dynamic acquisition was measured, and projection data were assessed for patient motion and motion artifacts. Patient motion was classified into various levels, and motion artifacts on SPECT images were visually examined.

Results: The distances of heart motion without and with the RRB were 15.4 ± 5.3 and 7.5 ± 2.3, respectively. Compared with the control group, the RRB group had a lower frequency of heart motion based on the projection data, particularly in terms of creep and shift motion. The RRB group had a significantly lower incidence of motion artifacts on SPECT images than the control group.

Conclusions: The RRB substantially reduced specific types of motion, such as shift and creep, and had a low influence on bounce motion. However, it could effectively suppress respiratory-induced heart motion and reduce motion artifacts on myocardial perfusion SPECT, thereby emphasizing its potential for improving image quality.

## Introduction

Phantom experiments and clinical studies have investigated the impact of image degradation caused by heart motion on myocardial perfusion single-photon emission computed tomography (SPECT) [1]. Based on the fraction and magnitude of heart motion, SPECT images may show ventricular wall discontinuities, non-anatomic perfusion defects, hot spots, and distorted ventricular shapes or hurricane signs [2]. Thus, projection data without patient motion should be acquired. However, several factors, including heartbeats, respiratory motion, upward creep after exercise loading, psychological factors due to anxiety, and shoulder pain, cause motion artifacts during acquisition. Previous studies have classified the types of vertical motion in the heart as repeated upward and downward bounce, shift (upward or downward shift without return motion), creep (continuous gradual upward or downward motion in either direction), or a combination of these, with bounce accounting for approximately 80% of cases [3]. Previous studies based on visual observation of projection data reported that patient motion was observed in 25%-36% of myocardial SPECT scans [4-6]. Approximately 80% of respiratory motion measurements via visual tracking of markers on the body surface showed >6 mm of vertical motion, with a maximum of 37 mm in a previous study [7]. Other reports show that respiratory-gated acquisition can improve the decrease in the inferior wall and septum uptake caused by respiratory motion [1].

Although patient instructions regarding breathing and body motion can be effective, the heart motion is challenging to completely eliminate. Therefore, previous studies have assessed body motion compensation and deep inspiration breath-hold acquisition with respiratory triggering [1]. Body motion compensation requires laborious processing, and breath-hold acquisition causes a burden on the patient and prolongs the examination duration. Furthermore, patients cannot hold their breath repeatedly in the exact inspiratory position without respiratory monitoring. Therefore, in coronary magnetic resonance angiography, the use of an abdominal belt has improved scanning efficiency [8]. In respiratory monitoring scanning, although an abdominal belt was effective in reducing imaging time, it only reduced the distance of liver motion by 35% (from 14.0 to 9.1 mm) in the vertical direction among Japanese patients. Therefore, an inexpensive device focusing on the suppression of respiratory motion of internal organs was developed. The current study aimed to evaluate the efficacy of the novel respiratory motion reduction block (RRB) device in suppressing the distance of vertical motion of the heart using dynamic images and projection data. Moreover, the role of the device in decreasing the incidence of artifacts on SPECT images was validated.

## Materials and methods

RRB method

**Figure 1:**
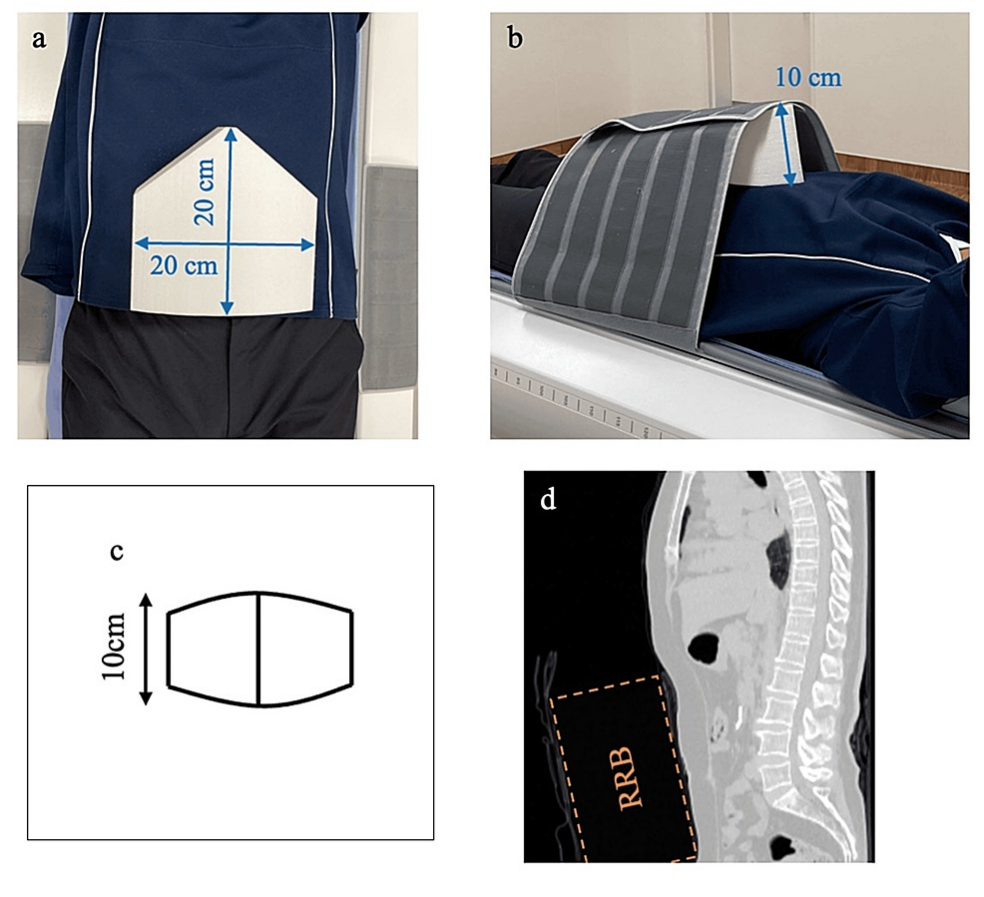
The respiratory motion reduction block (a) A pentagonal styrene foam device with a width of 20 cm and a length of 20 cm. (b) The RRB was tightly wrapped using the belt of the bed. (c) View of the RRB from the apex. (d) Computed tomography sagittal image of a female of Japanese standard proportions above 70 years old. RRB: Respiratory motion reduction block.

The RRB (self-made) was wrapped on the patient’s abdomen using a belt attached to the bed of the gamma camera system to effectively suppress the heart motion, which is associated with respiratory motion (Figure 1). RRB is a pentagonal Styrofoam device with a height of 20 cm, width of 20 cm, thickness of 10 cm, and a bulge at the center. As the thorax is challenging to compress because of the ribs, a convex shape that matches the shape of the hypochondrium is essential to avoid the ribs and suppress diaphragmatic motion. Moreover, Japanese patients are more likely to have an abdomen that is more concave than the chest while in the supine position [9,10]. Thus, with sufficient thickness, RRB should be higher than the chest to compress the abdomen with the belt. The device had a convex center to maximize the effect of abdominal compression. RRB was made of styrene foam to ensure that the scattering and absorption of gamma rays were negligible. To effectively suppress the heart motion, the convex part of RRB was aligned with the patient’s hypochondrium. Then, the patient's abdomen was compressed with the RRB in deep exhalation using a fixed belt.

Study population

We retrospectively evaluated 91 patients who underwent myocardial perfusion SPECT at our institution between June and December 2022 (Table 1). Patients with suspected or known coronary artery disease underwent the one-day stress­-rest or rest-delayed rest myocardial perfusion SPECT. As only adenosine-loaded or resting tests were included in our study, the heart motion caused by upward creep after exercise could be excluded. The control group comprised 28 patients who underwent SPECT without RRB. The RRB group included 63 patients who underwent SPECT with RRB. The study protocol was approved by the ethics committee of our institution (approval no.: 727). Due to the study’s retrospective design, informed consent was obtained from the patients in the form of opt-out.

**Table 1:** Characteristics of the patients P-values of <0.05 were considered statistically significant. RRB: Respiratory motion freeze; S–R: Stress–rest; E–D: Early–delay; BMI: Body mass index.

	Control group (n = 28)	RRB group (n = 63)	P-value
Male/female	16/12	45/18	0.181
S–R/E–D	25/3	58/5	0.698
Age (years)	70.7	71.5	0.628
Height (cm)	157.0	159.5	0.161
Weight (kg)	59.9	62.0	0.270
BMI (kg/m^2^)	24.2	24.3	0.432

Image acquisition and processing

All patients underwent scanning in two phases within one day: early (stress or rest) and delayed (rest). For all stress tests, adenosine (PDRadiopharma Inc., Tokyo, Japan) was administered intravenously at a rate of 120 μg/kg/min for 6 min without low-level exercise. Technetium 99m sestamibi (^99m^Tc-sestamibi) (PDRadiopharma Inc., Tokyo, Japan) was administered 3 min after the start of adenosine administration. Scanning based on the rest/rest protocol was performed at 1 and 3 h after the administration of ^99m^Tc-sestamibi at a dose of 259-370 MBq and light meal intake. Scanning based on the stress-rest protocol was performed 45-60 min after the administration of 99mTc-sestamibi at a dose of 259 and 740 MBq for the stress and rest test, respectively. The patients commonly had a light meal after each of the two injections, and the interval between the stress and rest injections was at least 2 h.

Symbia Evo equipped with low-energy high-resolution collimators (Siemens Healthineers, Erlangen, Germany) was the gamma camera used in this study. In the RRB group, the following parameters were used: 256 × 256 matrix (2.4 × 2.4 mm^2^ pixel), 1 s/frame × 60 frames anterior dynamic acquisition with and without RRB before the stress or rest SPECT. The SPECT acquisition parameters were as follows: energy window, 140 keV ± 7.5%, 128 × 128 matrix, ×1.45 zoom (3.3 × 3.3 mm^2^ pixel), 25-35 s/view, 72 views/360°, and neighboring elliptical orbit. However, as RRB was used in the RRB group, the acquisition orbit became separated from the patient only at the anterior aspect even though it was set to the neighboring elliptical orbit.

Dynamic images were denoised using the Pixon method (Planar Processing, Siemens Healthineers, Erlangen, Germany) with a blend ratio of 30% [11]. SPECT images were reconstructed using Flash3D (including resolution compensation) with nine subsets, 10 iterations, and a post-Gaussian filter with full width at a half maximum of 13 mm [12]. No attenuation correction, scatter correction, and motion correction were performed.

Assessment of patient motion and motion artifact

**Figure 2:**
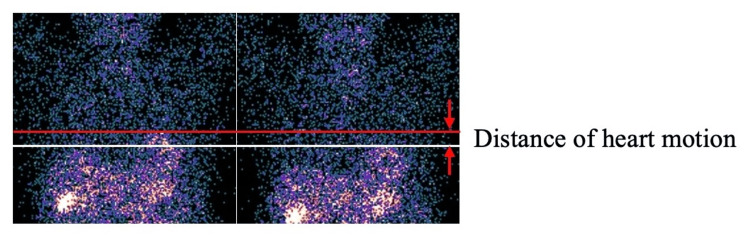
Measurement of the distance of heart motion Dynamic images were displayed using a cinematic display. Lines were set and confirmed at the end-inspiration (white) and end-expiration (red) phases, and the distance between the lines was measured. Pixon denoising was not performed on the images shown in this figure.

Using dynamic images, the distance of heart motion during scanning was measured while on cinematic display. Lines were set at the upper edge of the heart on end-inspiration and end-expiration, and the distance between the lines was measured at the maximum magnification, after confirmation by cinematic display (Figure 2). Moreover, all projection data on the degree of heart motion were assessed by observing cinematic displays, sinograms, and linograms. The degree of patient motion was classified into four levels, which were as follows: absent, mild, moderate, and severe. The types of patient motion were classified as vertical bounces, shifts, creep, and complex motion. The degree of motion artifact on SPECT images was also visually assessed as absent, mild, moderate, and severe. Visual classification was performed twice by an expert observer at intervals of at least two weeks, and another expert observer determined the cases with different classification results.

Statistical analysis

All data except on the nominal scale with a non-normal distribution were analyzed with the Shapiro-Wilk test. The nominal scale was tested with the chi-square test. Paired and unpaired values were examined with the Wilcoxon signed-rank test and Mann-Whitney U test, respectively. The correlations between age and weight as well as the heart motion in the control and RRB groups were determined by calculating Spearman’s rank correlation coefficients. Statistical analyses were performed using the SPSS software, version 27 (IBM Corp., Armonk, NY). P-values of <0.05 were considered statistically significant.

## Results

**Figure 3:**
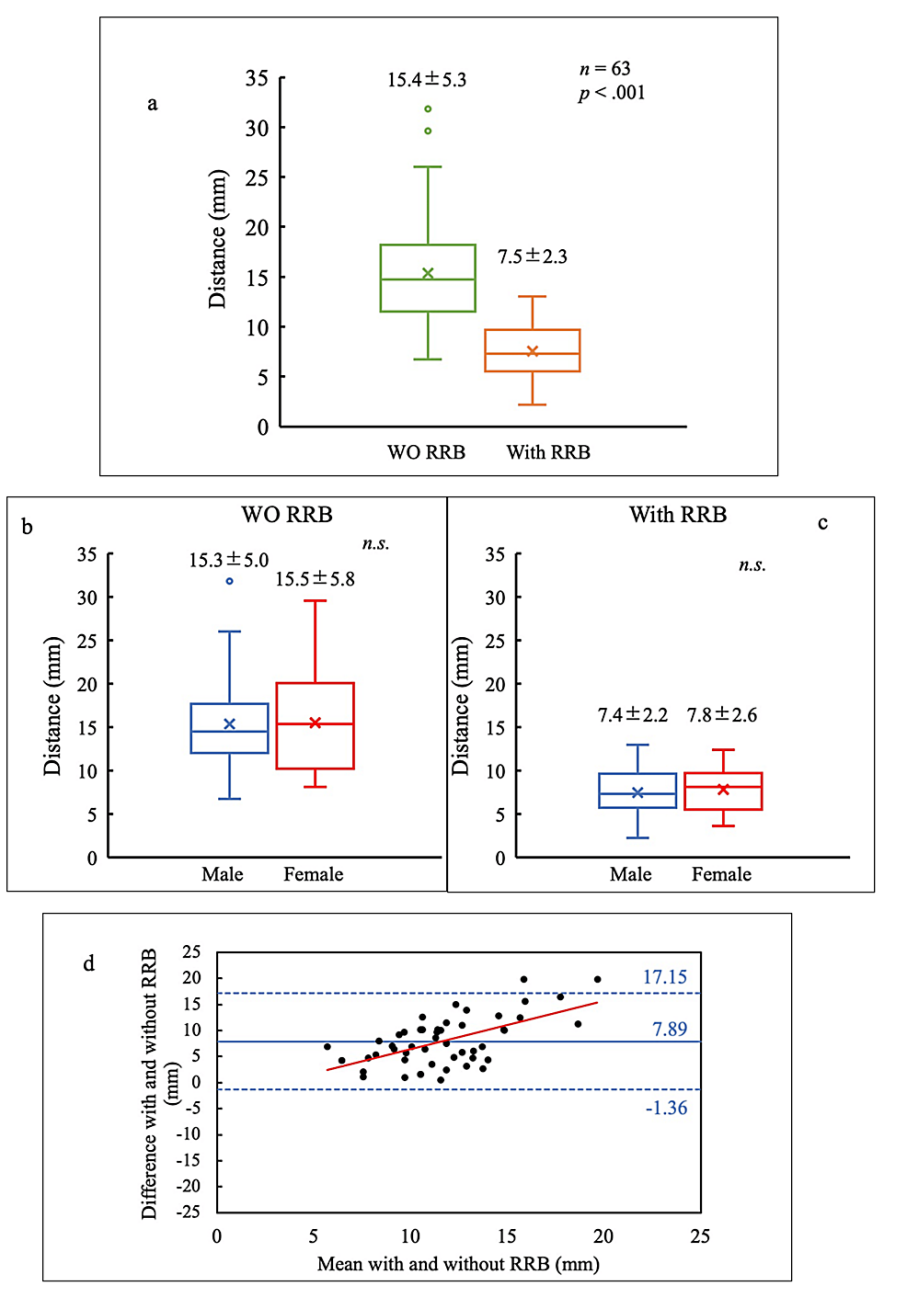
Box plots and Bland–Altman plots of heart motion distance (a) Comparison of scanning with and without respiratory motion reduction block (RRB). (b) Comparison between male and female patients without the use of RRB. (c) Comparison between male and female patients with the use of RRB. (d) Bland–Altman plots for comparing heart motion distance with and without RRB. Solid and dashed blue lines show the mean difference and 95% confidence interval, respectively.

No patient complained that they could not tolerate the wrapping of the RRB during the scanning. The distances of heart motion during dynamic acquisition without and with RRB were 15.4 ± 5.3 mm (range: 6.7-31.8) and 7.5 ± 2.3 mm (range: 2.2-13.0) respectively (p < .001; Figure 3, Panel a). If RRB was not used, the mean distances of heart motion were 15.3 ± 5.0 mm (range: 6.7-31.8) in men and 15.5 ± 5.8 mm (range: 8.1-29.6) in women. If RRB was used, the mean distances of heart motion were 7.4 ± 2.2 mm (range: 2.2-13.0) in men and 7.8 ± 2.6 mm (range: 3.6-12.4) in women. Hence, the results did not significantly differ between male and female patients (Figure 3, Panels b and c). Figure 3 (Panel d) shows the Bland-Altman plot comparing each patient who underwent dynamic imaging with and without RRB. The 95% confidence interval of the difference between scanning with RRB and scanning without RRB was 6.5656-9.2226 with a fixed bias (p < .001). In addition, there was a proportional bias between scanning with RRB and without RRB (p < .001).

**Figure 4:**
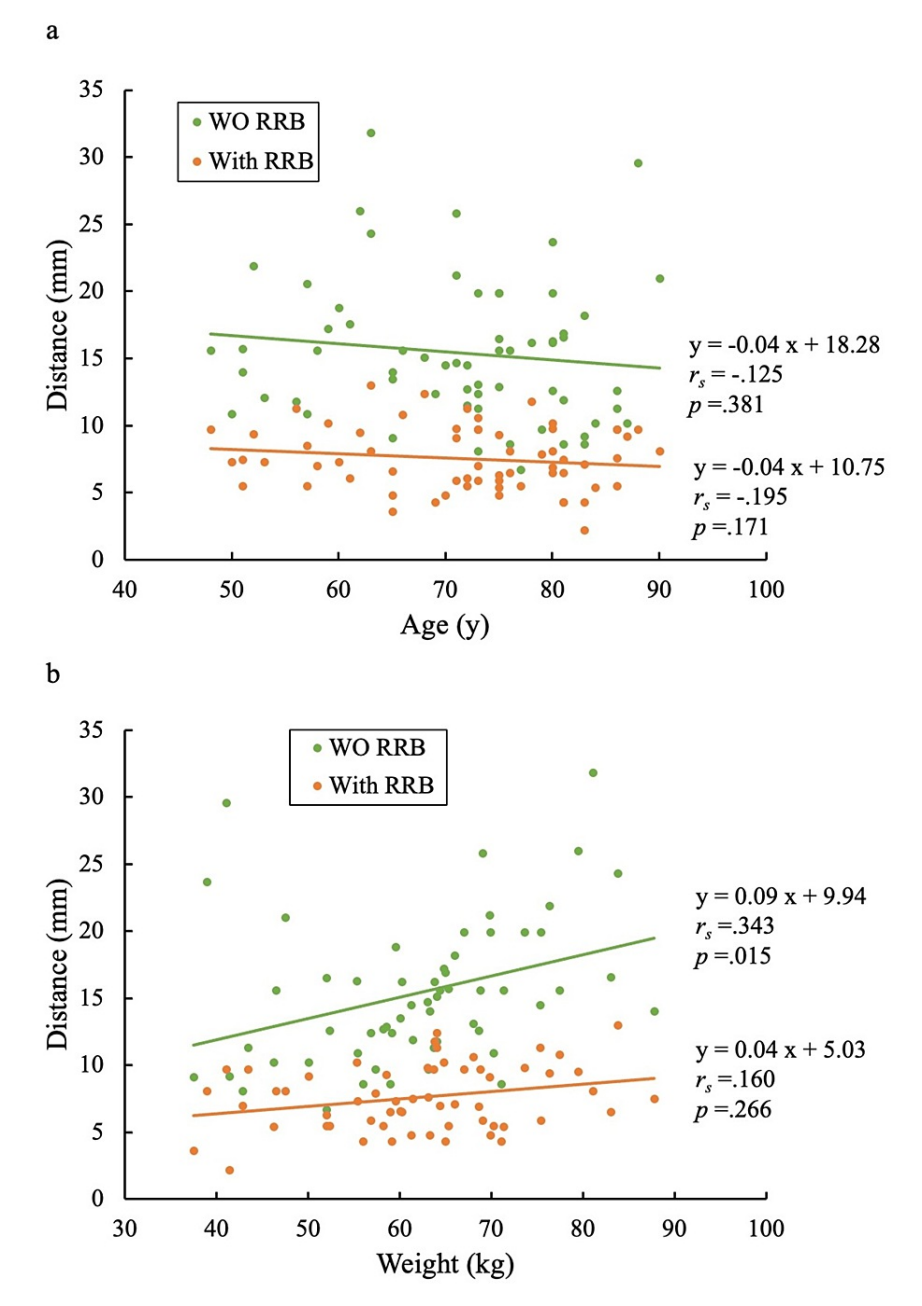
Scatter plots for age (a) and weight (b) versus the distance of heart motion WO RRB: Without respiratory motion reduction block.

There was no association between the distance of heart motion and age, with or without RRB (Figure 4, Panel a). Without RRB, the distance of heart motion was found to be associated with body weight (rs = .343); however, with RRB, there was no association between the distance of heart motion and body weight (Figure 4, Panel b). 

**Figure 5:**
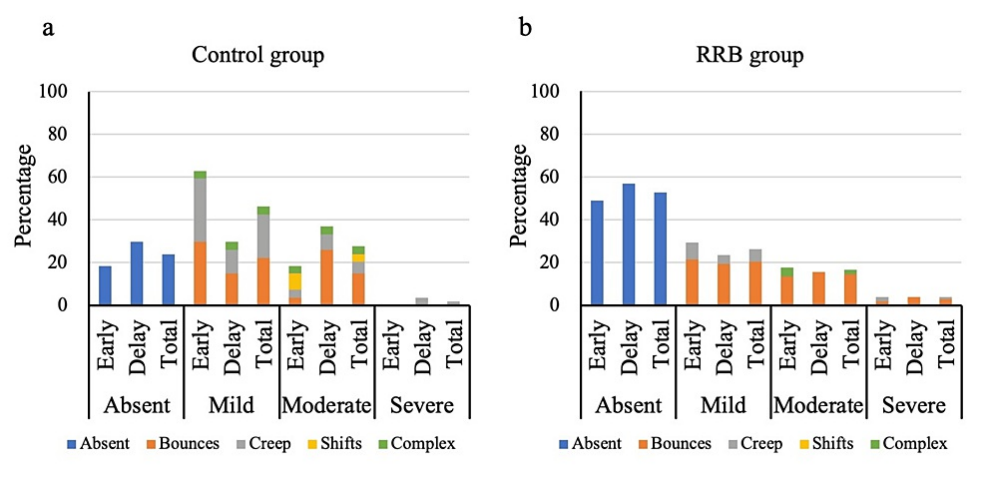
Visual assessment of patient motion based on the projection data The respiratory motion reduction block group (b) had a lower incidence of patient motion than the control group (a).

Figure 5 shows the incidence of the degree and types of motion based on the projection data. The RRB group had a significantly lower degree of patient motion than the control group (p = .011). During the delayed scan, the RRB group had a significantly lower degree of patient motion than the control group (p = .032). Although insignificant, the degree of patient motion during the early scan was lower in the RRB group than in the control group (p = .143). The degree of patient motion during delayed scanning was lower than that during early scanning in the control (p = .331) and RRB (p = .862) groups. However, the results did not significantly differ. The control group was more likely to present with creep and shift than the RRB group.

**Figure 6:**
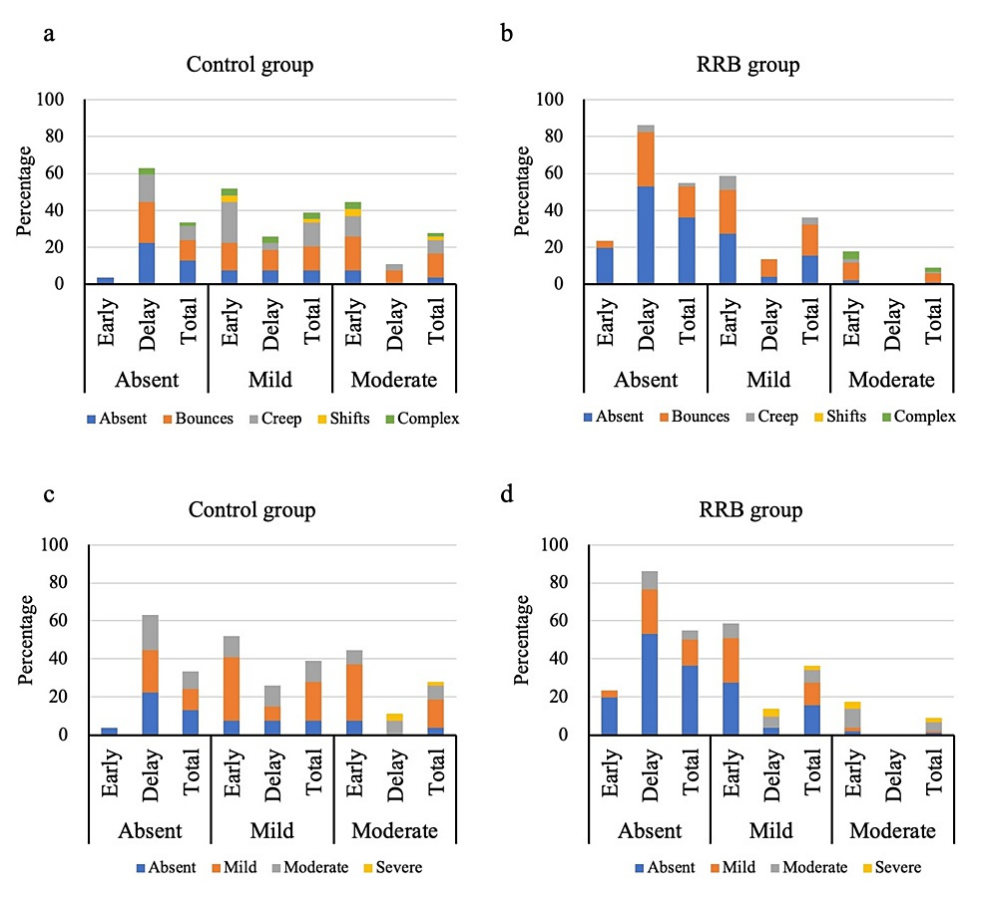
Visual assessment of motion artifacts on single-photon emission computed tomography (SPECT) images Association between the type (a, b) and degree (c, d) of motion based on the projection data versus motion artifacts on SPECT. No case was classified as "severe" artifacts.

Figure 6 shows the incidence of artifacts on SPECT images according to the degree and type of patient motion between the control and RRB groups. No cases were classified as severe artifacts in all SPECT images. The RRB group had a significantly lower incidence of motion artifacts on SPECT images than the control group (p = .002). The incidence of motion artifact on delayed SPECT was less than that on early SPECT in the control (p < .001) and RRB (p < .001) groups. The incidence of mild artifacts was similar between the control and RRB groups.

## Discussion

Vertical heart motion is one of the causes of artifacts, and its influence on diagnostic accuracy has been recognized. Thus, this study focused on the suppression of respiratory motion. We developed RRB to suppress the heart motion due to respiration and assessed its usefulness. Results showed that the distance of respiratory motion during projection data acquisition may be reduced to half. Hence, RRB may significantly reduce the incidence of artifacts on myocardial perfusion SPECT and reduce image distortion due to motion in the projection data.

RRB significantly reduced the distance of upward and downward heart motion during SPECT acquisition by significantly reducing the incidence of shifts and creep based on the projection data. As no exercise stress was performed in our study, RRB may have reduced the incidence of shift and creep due to not only respiratory motion but also body motion. Due to the prolonged SPECT acquisition duration, abdominal compression may have continued mild tension and reduced body motion. Our study revealed that heavier patients had a greater heart motion without RRB. However, neither age nor gender had any effect on the distance of the heart motion. In addition, if the distance of the heart motion is greater, the effect of RRB is more significant. In their study on the suppression of respiratory motion of organs in coronary magnetic resonance angiography, Ishida et al. [8] reported that the upward and downward motion of the liver caused by respiration was 14.0 mm in Japanese patients, and our results showed that the vertical heart motion was 15.4 mm, which is almost the same as their results. Their abdominal belt for respiratory motion suppressed the distance by 35%. However, RRB was more effective as it could suppress the distance by >50%.

In a previous study, the efficacy of abdominal belts in Japanese patients with a significantly smaller abdominal circumference was lower than that in British patients with a significantly larger abdominal circumference [8]. Nonetheless, the difference was not significant. Our results showed more respiratory motion suppression, and this might be attributed to the fact that RRB can compress the hypochondrium, which is closer to the diaphragm. If an abdominal belt was used to suppress the hypochondrium in Japanese patients, whose abdomen is often concave when in the supine position, the effect can be reduced by the ribs. Without RRB, not even the subtlest motion was detected during the two SPECT acquisitions in 24% of patients, which is clearly less than that in previous studies [5,6]. The results using visual tracking of body surface motion show that motion greater than 6 mm during SPECT acquisition was detected in 80% of the patients, which is similar to our results [7]. This might be caused by the fact that our facility used a 128 × 128 matrix and neighboring elliptical orbit for acquisition, thereby allowing the detection of a subtle motion compared with previous studies. Although the organs and target patients are different, we believe that our results are consistent with the results for organ motion distance in MRI images [8], which have much higher spatial resolution than gamma camera images and showed almost the same results as ours. Therefore, we believe that the validity of our results is supported by their study [7,8].

Other studies have focused on investigating the type, timing, and magnitude of motion in projection data and their contributing factors [2-6,13,14] as well as compensation for data with motion [7,15,16]. In generally used dual-detector cameras, artifacts are generated by shifts of two or more pixels, regardless of motion timing [3]. Therefore, controlling shift motion may be an important imaging technique to improve the diagnostic accuracy of myocardial perfusion SPECT. The use of RRB significantly reduced the incidence of shift and creep. However, it could not significantly reduce the incidence of bounce. Due to the high patient burden and prolonged examination duration, breath-hold imaging is not feasible, and motion correction is a laborious procedure. Respiratory motion is also known to potentially cause significant loss in image quality, which is not corrected with automated correction of inter-projection motion algorithms. Hence, it is important to suppress respiratory motion as much as possible [2]. This concern was addressed in this study with the proposed device to suppress respiratory motion. The use of RRB could be a cost-effective and simple technique for reducing substantial artifacts without prolonging the acquisition duration.

Although it is often difficult to eliminate a bounce completely unless the patient holds breathing in a similarly repeated fashion, a mild bounce may not be significantly related to substantial artifacts on SPECT images [5]. Although the motion of ≥2 pixels can generate clinically important artifacts [3], our results could not determine an evident difference based on the magnitude of the motion alone. The timing of motion may be strongly related to the incidence of artifacts, even in dual-detector cameras [5]. The incidence of artifacts was significantly higher in early scans than in delayed scans for the control and RRB groups, which is consistent with previous studies. This might be due to anxiety about undergoing early scans that were never experienced before [6]. Psychological discomfort is a factor affecting patient motion during SPECT acquisition in female patients [14]. However, our results showed no sex difference. In the visual evaluation of the projection data, we expected that age-related shoulder pain and cognitive decline could result in more frequent body motion during SPECT acquisition. Nevertheless, these factors were not related to body motion. This might be attributed to the fact that the arms were placed on the armrests and secured with belts, and the remaining time and other information were provided to the patients during scanning.

Abdominal breathing, also known as diaphragmatic breathing, decreases chest wall motion but causes the heart to move significantly upward and downward with the vertical motion of the diaphragm. Thoracic breathing involves expansion and contraction of the chest wall, causing the thorax to move anteriorly and posteriorly. However, the distance is less than that of vertical motion [17]. The preference for either thoracic or abdominal breathing in deep breathing differs per individual. Nonetheless, in general, thoracic breathing is predominant during shallow breathing in women. Our results showed no significant difference in the distance of heart motion with or without RRB between male and female patients. In myocardial perfusion SPECT, there has been almost no research on approaches for reducing artifacts via the suppression of respiratory motion, although respiratory-gated or respiratory motion correction SPECT has been studied [1]. Artifacts could be further reduced by combining motion correction with the use of RRB, which can be a major step toward reducing respiratory motion artifacts in myocardial perfusion SPECT.

This study had several limitations. For example, whether the defect artifact was indeed an artifact was not confirmed via imaging modalities, such as coronary angiography. It was difficult to perform further testing in patients with a normal diagnosis (with or without artifacts). However, at least the motion in the projection data was definitely reduced, and we believe that the motion artifacts in the SPECT images can be reduced accordingly. The incidence of artifacts may not be directly comparable to that in previous studies because of differences in pixel size and reconstruction methods [18]. We believe that our study was conducted under stringent conditions of pixel sizes of 2.4 and 3.3 mm for dynamic and SPECT acquisition, respectively. The impact of differences in abdominal versus thoracic breathing was not determined. Image quality degradation caused by the departure of the patient’s thorax from the detector caused by RRB was not assessed. Future phantom experiments should be conducted to verify whether image quality degradation occurs. Although it was not possible to distinguish whether the heart motion except for the bounce was caused by respiratory motion or body motion, it seems that body motion was also reduced by fixing the body with a belt and fixing the arms to the armrests.

## Conclusions

RRB was developed, and its efficacy in suppressing respiratory motion was assessed. RRB significantly reduces heart motion during SPECT acquisition, leading to fewer shifts and creeps in projection data. Heavier patients have greater heart motion, and patients with greater motion are more effective in motion suppression by RRB. The use of RRB reduces motion artifacts on myocardial perfusion SPECT, which is a simple technique for enhancing image quality without extending acquisition time.
